# *Allium sativum* L. Improves Visual Memory and Attention in Healthy Human Volunteers

**DOI:** 10.1155/2015/103416

**Published:** 2015-08-13

**Authors:** Sara Tasnim, Parsa Sanjana Haque, Md. Sazzadul Bari, Md. Monir Hossain, Sardar Mohd. Ashraful Islam, Mohammad Shahriar, Mohiuddin Ahmed Bhuiyan, Muhammad Shahdaat Bin Sayeed

**Affiliations:** ^1^Department of Pharmacy, University of Dhaka, Dhaka 1000, Bangladesh; ^2^Department of Pharmacy, Noakhali Science and Technology University, Noakhali 3814, Bangladesh; ^3^Department of Pharmacy, University of Asia Pacific, Dhaka 1209, Bangladesh; ^4^Department of Clinical Pharmacy and Pharmacology, University of Dhaka, Dhaka 1000, Bangladesh

## Abstract

Studies have shown that* Allium sativum* L. (AS) protects amyloid-beta peptide-induced apoptosis, prevents oxidative insults to neurons and synapses, and thus prevent Alzheimer's disease progression in experimental animals. However, there is no experimental evidence in human regarding its putative role in memory and cognition. We have studied the effect of AS consumption by healthy human volunteers on visual memory, verbal memory, attention, and executive function in comparison to control subjects taking placebo. The study was conducted over five weeks and twenty volunteers of both genders were recruited and divided randomly into two groups: A (AS) and B (placebo). Both groups participated in the 6 computerized neuropsychological tests of the Cambridge Neuropsychological Test Automated Battery (CANTAB) twice: at the beginning and after five weeks of the study. We found statistically significant difference (*p* < 0.05) in several parameters of visual memory and attention due to AS ingestion. We also found statistically nonsignificant (*p* > 0.05) beneficial effects on verbal memory and executive function within a short period of time among the volunteers. Study for a longer period of time with patients suffering from neurodegenerative diseases might yield more relevant results regarding the potential therapeutic role of AS.

## 1. Introduction

*Allium sativum* L. (AS) (family Amaryllidaceae), commonly known as garlic, is one of the most widely quoted herbs found in the old medical literatures mainly for its medicinal potentials. Its use as a medicinal herb dates back to about 1550 BC, as indicated by its citation in the* Ebers Papyrus* [1]. In ancient time, AS was used as a preservative for food and as an aid to digestion and respiration to provide energy and elevate depression and was prescribed for animal bites, arthritis, and convulsions [2].

AS, as an ingredient in normal diets, has been considered safe for human consumption and has wide variety of beneficial applications [3]. It has been reported to reduce the risk of cardiovascular disease progression upon its regular consumption [4]. Previous studies also demonstrated that AS has a potent effect on lowering the plasma cholesterol level in animals as well as humans [5].* S*-Allyl cysteine (SAC) found in AS was found to exert neuroprotective effects by attenuating 1-methyl-4-phenylpyridinium induced neurotoxicity in the striatum of mice [6]. SAC (L-isomer) was also suggested to preserve the ischemic neurovascular units mainly in cerebral region [7]. Several antioxidants present in AS were reported to be effective in scavenging reactive oxygen species (ROS) and inhibiting low density lipoprotein (LDL) oxidation [8]. Studies reported that AS acts as an effective antioxidant in preventing endothelial cell damage as it inhibits lipid peroxide formation and also increases the activity of superoxide dismutase, catalase, and glutathione peroxidase enzymes [9]. Even though excessive AS consumption has been reported to cause dermatitis [10, 11], exfoliation [12], damage of the gastric epithelia [13], hepatomegaly, splenomegaly, adrenomegaly, and decreased count of erythrocytes [14], generally, normal consumption of AS is considered to be safe for humans.

Previous studies have shown that AS improves the spatial memory deficit in senescence accelerated mouse [15]. A study by Sadeghi et al. [16] has suggested that AS protects hippocampal neurons against lead-induced neural damage in rat offspring. Studies by Mukherjee and Banerjee [17] have shown that extract of AS prevents memory impairment. Moreover, the antioxidant, antiapoptotic, and antiatherogenic properties of AS contribute to the protection of neurons [18]. A study by Peng et al. [19] has shown that AS protects amyloid-beta peptide-induced apoptosis* in vitro* and its active compound, SAC, plays significant role in preventing oxidative insults to neurons and synapses and thus prevents Alzheimer's disease progression [20, 21]. The study by Chen et al. [22] suggests that neuroprotective effect of AS against traumatic brain injury is provided by anti-inflammatory and antioxidant activities mediated via endothelial nitric oxide synthase pathway. These studies form the basis of our investigation regarding the putative role of AS in memory formation, retrieval, and memory degeneration prevention.

To the best of our knowledge there is no study conducted regarding the effect of AS consumption by healthy human volunteers on visual memory, verbal memory, attention, and executive function in comparison to control healthy volunteers taking placebo. To address this we used CANTABeclipse to find the effect of AS on human visual memory and new learning, immediate and delayed verbal information, visual sustained attention, rule acquisition and reversal, retention of spatial information and manipulation of remembered items in working memory, spatial planning, and working memory.

## 2. Methods and Assessment

### 2.1. Participants

Twenty healthy volunteers (14 males and 6 females) were randomly recruited, with ages ranging from 21 to 23 years, participating in the present study. One of the volunteers found the experiment tiresome and so did not participate in the second session. Written informed consent was obtained from each volunteer prior to study. The volunteers completed a set of medical health questionnaires to evaluate their health conditions for the suitability of the study. Eight of the selected volunteers were habitual smokers. Subjects were asked to avoid caffeine before 12 hours of the study. The study (code number UAPSBS201401) was conducted in accordance with the International Conference of Harmonization (ICH) for Good Clinical Practice (GCP) and in compliance with the Declaration of Helsinki and its further amendments [23].

### 2.2. Preparation of Capsules

AS (garlic) skins were peeled off and the bulbs were sliced into thin pieces. Slices were sun-dried for three days (exposure to sunlight ~40 hours) by keeping them within a glass box. The sun-dried slices were crushed with the help of mortar and pestle to form a paste. The paste was immediately transferred on a cotton cloth which absorbed the moisture as well as the oil. Then it was further dried in sunlight for one and half days (~20 hours). Finally the dried paste was reduced to fine powder using a dry blender. The powdered garlic was passed through a stainless steel screen (mesh size #20) and then dried in a tray drier at 35°C for 10 minutes. CAB-O-SIL (fumed silica) was added to improve fluidity and resist moisture. The powder was filled into empty hard gelatin capsule shells (size #0) using a handheld capsule filling machine. The whole process was vigorously monitored under the direct supervision of an experienced pharmacist. The above process was undertaken in a local GMP compliant company (Amicon Laboratories, Bangladesh). It was ensured that each of the capsules contained 400 mg powdered garlic. Husk of isabgol (*Psyllium* seed husk) was used as placebo and capsules were prepared in a similar fashion. Both the garlic and isabgol were identified and preserved with specifications under study code with the supervision of institutional pharmacist.

### 2.3. Treatment and Design

**Figure 1:**
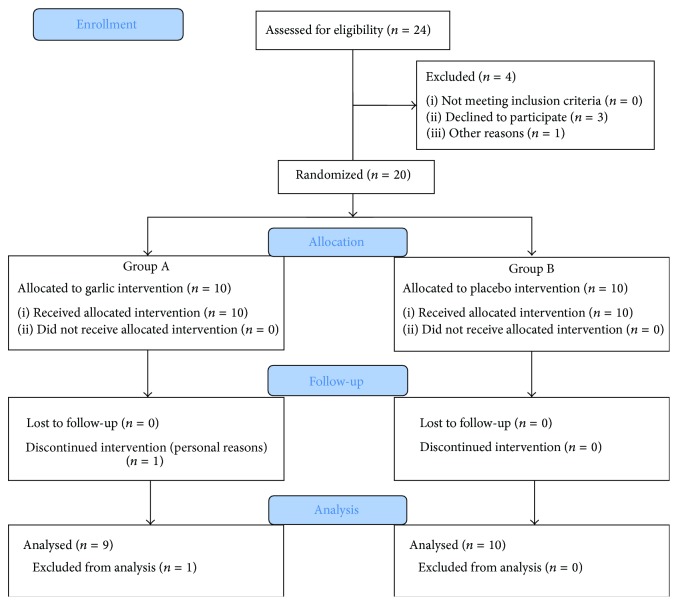
CONSORT 2010 flow diagram for the study design.

The study was conducted over five weeks. Volunteers were randomly divided into two groups (Figure 1). One group (group A) received one 400 mg AS capsule twice daily in the morning and the evening for five weeks. The dose was selected based on previous reports [24–26]. The second group (group B) received placebo in a similar manner for the same period of time. Placebo and garlic capsules had the same color, texture, size, shape, and smell. All the volunteers were assessed for baseline data to measure the condition of memory, attentiveness, and cognition before the administration of the first dose of either AS capsules or placebo. The sequences of the tests administered were kept constant for all the volunteers and were completed between 1400 and 1700 h. The volunteers were assessed for all the parameters measured at two time points: baseline and after the fifth week of capsule administration. The instructions for the tests were explained to the volunteers before conducting the study. All volunteers were kept blind about garlic or placebo and the code numbers and the group allocation were only revealed after the assessment of the last subject. All volunteers were instructed to call the study center in case of any adverse effect during the study. Volunteers had the opportunity to withdraw from the study at any time. All the volunteers were contacted at definite intervals to ensure that they took the capsules regularly.

### 2.4. Assessment

The volunteers were asked to sit for a series of 6 computerized neuropsychological tests of the Cambridge Neuropsychological Test Automated Battery (CANTAB) which is a widely used tool for assessing cognitive function, memory, and attention.

#### 2.4.1. Visual Memory Test

*Paired Associates Learning (PAL)*. PAL assesses visual memory and new learning. The volunteers were shown up to 12 patterns by opening 12 individual boxes in random order on the screen. Each pattern then appeared in the center of the screen and they had to identify the box containing the particular pattern. They could proceed to the next stage only when all the patterns were correctly identified. Outcome measures include the following.

- PAL total errors adjusted: the total errors made in all stages along with an adjustment for each stage not attempted due to previous failure.
- PAL mean errors to success: the mean errors made before a stage was successfully completed.
- PAL mean trials to success: the total trials required to locate all the patterns correctly.
- PAL stages completed: the final number of stages completed by the volunteers.
- PAL total errors (8 shapes, adjusted): the total errors made in the 8-stage pattern with an adjustment for those who have not reached this stage.

#### 2.4.2. Verbal Memory Test

*Verbal Recognition Memory (VRM)*. VRM assesses immediate and delayed verbal information. The volunteers were shown a list of 18 words and were asked to remember as many of them as they could. They also had to recognize those words when presented along with a list of distractor words on the screen. After a delay of 20 minutes, the recognition phase was repeated again. Outcome measures include the following.

- VRM free recall total correct (immediate)—total number of words correctly recalled.
- VRM free recall total novel words (immediate)—total number of new words recalled.
- VRM recognition total correct (immediate)—total number of words correctly recognized among the distractor words.
- VRM total false positives (immediate)—total number of distractor words recognized as part of the main list.
- VRM recognition total correct (delayed)—total number of words correctly recognized among the distractor words 20 minutes after presentation of the list.

#### 2.4.3. Attention Test

*Rapid Visual Information Processing (RVP)*. RVP is a test of visual sustained attention. The volunteers had to recognize a sequence of digits from a pseudorandom order of digits that appeared in the center of the screen. The digits appeared at the rate of 100 digits per minute. After recognition, they had to depress a press pad. Outcome measures include the following.

- RVP A′—the probability to detect the target sequence.
- RVP B′—the probability to depress the press pad irrespective of the presence of the target sequence.
- RVP total hits—the number of occasions upon which the target sequence was correctly identified.

#### 2.4.4. Executive Function Tests

*(1) Intra-Extra Dimensional Set Shift (IED)*. IED is a test of rule acquisition and reversal. The volunteers were presented with two shapes and they had to identify the correct one by touching it on the screen. At a point the rules changed and they had to keep on choosing the correct pattern. Outcome measures include the following.

- IED total trials (adjusted)—the number of trials completed on all attempted stages with an adjustment for any stages not reached.
- IED total errors (adjusted)—efficiency in attempting the test.
- IED stages completed—total number of stages completed successfully.

*(2) One Touch Stockings of Cambridge (OTS)*. OTS is a test of spatial planning and working memory. The screen presents a set of balls in different pockets. The volunteers had to move the balls in minimum possible moves to make a similar arrangement as the computer in their minds and select the move number on the screen. Outcome measures include the following.

- OTS problems solved on first choice—the number of problems which were solved on the first choice.
- OTS mean choices to correct (5 moves)—the mean number of unique box choices that was made on 5-move problem to make the correct choice.
- OTS mean latency to first choice—the mean latency measured from the time the balls appeared on the screen until the box was touched.
- OTS mean latency to first choice (5 moves)—the mean latency measured from the time the balls appeared on the screen until the box was touched in case of the 5-move problem.
- OTS mean latency to correct (5 moves)—the mean latency measured from the time the balls appeared on the screen until the box was touched in case of the 5-move problem.

*(3) Spatial Working Memory (SWM)*. SWM assesses retention of spatial information and manipulation of remembered items in working memory. The screen presents a number of boxes with blue tokens hidden in them. The volunteers had to find the tokens and fill up an empty bar on the side of the screen. However, the trick was not to visit the box in which the token had already been found. The number of token boxes gradually increases. Outcome measures include the following.

- SWM between errors—the number of times the volunteer visited the box in which the blue token had already been found.
- SWM strategy—the number of times the participant begins a search with the same box for 6- and 8-box problems.

### 2.5. Statistical Analysis

Results were analyzed independently for each test. Poisson regression was employed to identify whether group has any significant difference over the time. To estimate the parameters of the model, we employed generalized estimating equations as over the time the responses are associated with employing *R*. To find the difference between placebo and treatment groups we checked normality assumption and employed statistical tests that appropriate. Repeated measure ANOVA was employed to observe between and within effect by using IBM Statistics 21. Repeated measure multivariate analysis was performed to find the effect of AS over four-week time period. Chi-square test was performed for demographic data between groups. *p* < 0.05 was considered statistically significant.

## 3. Result

### 3.1. Test of Visual Memory

Repeated measure multivariate analysis shows that difference between group A and group B on PAL over five-week study period was not statistically significant, *F*(5,13) = 2.357, *p* = 0.113, and *η* ^2^ = 0.320, but univariate tests indicated that AS has significant effect on four out of five tests of PAL; *F*(1,17) = 6.899, *p* = 0.018, and *η* ^2^ = 0.289 for PAL total errors (adjusted); *F*(1,17) = 6.899, *p* = 0.018, and *η* ^2^ = 0.289 for PAL mean errors to success; *F*(1,17) = 4.769, *p* = 0.043, and *η* ^2^ = 0.219 for PAL mean trials to success; and *F*(1,17) = 4.808, *p* = 0.043, and *η* ^2^ = 0.220 for PAL total errors (8 shapes, adjusted). Since all the subjects had the value of 5 for “PAL stages completed” at both time points, analysis of this parameter was not possible and therefore excluded from analysis (Table 1).

### 3.2. Test of Verbal Memory

Variation of VRM was not statistically significant (*p* < 0.05). Repeated measure multivariate analysis shows that difference between group A and group B on VRM over five-week study period was not statistically significant, *F*(5,13) = 0.474, *p* = 0.789, and *η* ^2^ = 0.154. Univariate tests also indicated that AS has no effect on all of the tests of VRM, *F*(1,17) = 0.034, *p* = 0.856, and *η* ^2^ = 0.002 for VRM free recall—total correct (immediate), *F*(1,17) = 1.607, *p* = 0.086, and *η* ^2^ = 0.002 for VRM free recall—total novel words (immediate), *F*(1,17) = 0.032, *p* = 0.860, and *η* ^2^ = 0.002 for VRM recognition—total correct (immediate), *F*(1,17) = 0.067, *p* = 0.799, and *η* ^2^ = 0.004 for VRM recognition—total false positives (immediate), and *F*(1,17) = 0.198, *p* = 0.662, and *η* ^2^ = 0.012 for VRM recognition—total correct (delayed) (Table 1).

### 3.3. Test of Attention

The results for the attention test (RVP) did not show statistically significant variation (*p* < 0.05). Repeated measure multivariate analysis shows that difference between group A and group B on RVP over five-week study period was not statistically significant, *F*(3,15) = 2.449, *p* = 0.104, and *η* ^2^ = 0.333. Univariate test also indicated that AS has no effect on one of the tests of RVP, RVP B′′ with *F*(1,17) = 0.063, *p* = 0.805, and *η* ^2^ = 0.004. However, univariate tests indicated that AS has statistically significant effect on RVP A with *F*(1,17) = 15.392, *p* = 0.001, and *η* ^2^ = 0.475 and RVP total hits with *F*(1,17) = 17.704, *p* = 0.001, and *η* ^2^ = 0.510 (Table 2).

### 3.4. Executive Function Tests

#### 3.4.1. Intra-Extra Dimensional Shift

Variation of IED was not statistically significant (*p* > 0.05). Repeated measure multivariate analysis shows that difference between group A and group B on IED over five-week study period was not statistically significant, *F*(3,15) = 0.49, *p* = 0.695, and *η* ^2^ = 0.089. Univariate tests also indicated that AS has no effect on all of the tests of IED, *F*(1,17) = 0.665, *p* = 0.426, and *η* ^2^ = 0.038 for IED total trials (adjusted), *F*(1,17) = 0.628, *p* = 0.439, and *η* ^2^ = 0.036 for IED Total errors (adjusted), and *F*(1,17) = 0.625, *p* = 0.440, and *η* ^2^ = 0.035 for calmness IED stages completed, respectively (Table 3).

#### 3.4.2. One Touch Stocking of Cambridge

We did not find any statistically significant difference between groups A and B for OTS (*p* > 0.05). Repeated measure multivariate analysis shows that difference between group A and group B on OTS over five-week study period was not statistically significant, *F*(5,13) = 0.919, *p* = 0.499, and *η* ^2^ = 0.261. Univariate tests also indicated that AS has no effect on all of the tests of OTS: *F*(1,17) = 0.251, *p* = 0.623, and *η* ^2^ = 0.015 for problem solved on first choice, *F*(1,17) = 0.015, *p* = 0.903, and *η* ^2^ = 0.001 for OTS mean choices to correct (5 moves), *F*(1,17) = 0.010, *p* = 0.923, and *η* ^2^ = 0.001 for OTS mean latency to first choice, *F*(1,17) = 0.007, *p* = 0.934, and *η* ^2^ = 0.000 for OTS mean latency to first choice (5 moves), and *F*(1,17) = 0.861, *p* = 0.366, and *η* ^2^ = 0.048 for OTS mean latency to correct (5 moves) (Table 3).

#### 3.4.3. Spatial Working Memory

There were no significant difference between groups A and B for SWM (*p* > 0.05). Repeated measure multivariate analysis shows that difference between group A and group B on SWM over five-week study period was not statistically significant, *F*(2,16) = 0.201, *p* = 0.820, and *η* ^2^ = 0.261. Univariate tests also indicated that AS has no effect on all of the tests of SWM: *F*(1,17) = 0.427, *p* = 0.522, and *η* ^2^ = 0.025 for SWM between errors and *F*(1,17) = 0.100, *p* = 0.755, and *η* ^2^ = 0.006 for SWM strategy (Table 3).

## 4. Discussion

In the present study, we analyzed the effect of AS on human memory, attention, and executive function by running a series of neuropsychological tests on the volunteers before and after AS capsule administration. Improvements have been found in all the measures of the visual memory test (PAL) after the 5-week study period. The mean for all the measures PAL total errors (adjusted), PAL mean errors to success, PAL mean trails to success, and PAL total errors (8 shapes, adjusted) showed a significant decrease (*p* < 0.05) after AS administration from baseline results in group A. These results indicated that the volunteers in group A made fewer errors in locating the correct pattern in the right box. They also needed fewer attempts to locate the right box containing the correct pattern. On the other hand, the mean for the volunteers in group B decreased nonsignificantly after 5 weeks (*p* > 0.05). So it is evident that AS administration made an improvement in visual memory of humans, but this statistically significant improvement might be due to the fact that there was a large baseline difference between groups in visual memory although such difference was nonsignificant. However, no such improvements have been found in case of verbal memory test (VRM) after the 5-week study period. For group A, there was a characteristic increase in the mean of VRM free recall—total correct (immediate),VRM free recall—total novel words (immediate), VRM recognition—total correct (immediate), and VRM recognition—total correct (delayed), but these improvements were not statistically significant (*p* > 0.05). The mean of VRM recognition total false positives (immediate) showed an overall decrease from baseline results but was not statistically significant (*p* > 0.05). In case of group B, the values were nonsignificantly different after placebo intake (*p* > 0.05).

Another key observation was found in the measures of the attention test. The mean for RVP A′′ and RVP total hits showed a significant increase for group A after 5 weeks (*p* < 0.05). The result indicated that the volunteers, after receiving AS capsules, showed a greater tendency of discerning the target sequence correctly. So, it can be inferred that AS had an ameliorating effect on the attention of the volunteers. However, the mean for RVP B′′ nonsignificantly decreased after 5 weeks whereas, in the case of group B, there was an increase in the mean for all the measures, but it was not statistically significant (*p* > 0.05).

Nonsignificant changes were found in the executive function test, IED, for both groups of volunteers (*p* > 0.05). Similarly, the mean of the other two executive tests, OTS and SWM, also did not change significantly among and between the two groups (*p* > 0.05). So, consumption of AS capsule did not affect any prominent changes in the executive function among the volunteers.

Therefore, our key findings demonstrate the attention and memory enhancing properties of AS in human model. Given the age of the subject and duration of the study it is difficult to attribute the beneficial effects of AS on a single of underlying mechanism. However, we propose that in case of elderly people whose antioxidant and acetylcholine esterase activities have been compromised, AS might act in preventing or slowing the progression of memory degeneration via its antioxidant and acetylcholine esterase activities [27–29]. Since it has been found that increased oxidative stress can be linked to decline in memory in animal models [30, 31]. Generally, oxidative stress is caused by production of excessive amounts of free radicals ROS and RNS that exceeds the capacity of endogenous antioxidant defense system. So, it can be hypothesized that AS has significant antioxidant properties which modulated improvements in memory and attention. As mentioned earlier, SAC is one of the major components of AS. It gets metabolized in the body to cysteine, which is a direct precursor of endogenous antioxidant glutathione (GSH) [32]. Studies have reported that it directly scavenges free radicals and also restores cerebral acetylcholinesterase activity [32, 33] and, therefore, we propose extensive study with SAC and its derivatives for their possible effect in preventing or slowing the progression of neurodegeneration.

In this study period, the volunteers did not experience any adverse clinical symptoms. We measured systolic and diastolic blood pressure and weight but did not find any difference before and after study period (*p* values were 0.654, 0.543, and 0.876, resp.). This is in compliance with the fact that garlic is widely used as spice in different foods and has not been reported negatively for its adverse effect.

Considering the result obtained data from the CANTAB software and the statistical significance of the test results, it can be said that AS might improve visual memory and attention. Although expected changes (either increasing or decreasing) were observed in the test results of all of verbal memory tests and executive function tests, they were not statistically significant (*p* > 0.05). It is suggested that a study conducted using patients suffering from neurodegenerative diseases such as Alzheimer's disease for a longer period of time may yield statistically significant results for AS in memory, new learning, and attention. This study could be cited as evidence for AS as a natural memory protectant and people taking AS in their food as nutrient might slow the onset and/or progression neurodegenerative diseases.

## Supplementary Material


*Allium sativum* L. (Garlic) was sliced into pieces and then crushed with a mortar and pestle to form a paste. The paste was dried under the sun and then reduced to a fine powder using a dry blender. Dried garlic powder (400mg) was then filled into empty capsules with the help of a small-scale capsule filling machine. The prepared capsules were then taken by healthy human volunteers for 5 weeks. The volunteers participated in 6 computerized neuropsychological tests of the Cambridge Neuropsychological Test Automated Battery (CANTAB) at the beginning and end of the 5 weeks study period. The results showed statistically significant variation in visual memory and attention while statistically non-significant variation was found in executive function.

## Figures and Tables

**Table 1:** Memory tests.

Neuropsychological tests			Group A (*n* = 9)		Group B (*n* = 10)	
			Baseline	After 5 weeks	Baseline	After 5 weeks
Visual Memory Test	Paired Associative Learning (PAL)	PAL total errors (adjusted)^*∗*^	31 (22.65)	13.22 (8.23)	15.1 (9.78)	13.7 (10.4)
		PAL mean errors to success^*∗*^	6.2 (4.53)	2.64 (1.65)	3.02 (1.96)	2.74 (2.08)
		PAL mean trials to success^*∗*^	2.69 (0.81)	1.86 (0.51)	2.08 (0.6)	1.82 (0.37)
		PAL total errors (8 shapes, adjusted)^*∗*^	4.78 (5.43)	0.78 (1.3)	1.6 (2.5)	1.6 (2.99)

Verbal Memory Test	Verbal Recognition Memory (VRM)	VRM free recall—total correct (immediate)	11.11 (3.3)	12.89 (1.45)	11.2 (2.86)	12.7 (1.77)
		VRM free recall—total novel words (immediate)	0.11 (0.33)	0.44 (0.88)	0.6 (0.97)	0.4 (0.97)
		VRM recognition—total correct (immediate)	34.67 (0.71)	35.22 (0.67)	34.4 (1.96)	35 (1.7)
		VRM recognition—total false positives (immediate)	0.33 (0.5)	0.22 (0.44)	0.3 (0.67)	0.4 (0.97)
		VRM recognition—total correct (delayed)	34.44 (1.59)	34.78 (1.92)	34.1 (1.85)	33.4 (5.89)

The values are expressed as “mean (SD)” and *∗* indicates that variation is statistically significant (*p* < 0.05).

**Table 2:** Attention test.

Neuropsychological tests			Group A (*n* = 9)		Group B (*n* = 10)	
			Baseline	After 5 weeks	Baseline	After 5 weeks
Attention Test	Rapid Visual Information Processing (RVP)	RVP A^′*∗*^	0.91 (0.04)	0.94 (0.04)	0.93 (0.05)	0.94 (0.03)
		RVP B′′	0. 95 (0.39)	0.95 (0.07)	0.95 (0.05)	0.95 (0.07)
		RVP total hits^*∗*^	17.78 (3.87)	20.78 (4.44)	19.5 (5.49)	20.8 (3.55)

The values are expressed as “mean (SD)” and *∗* indicates that variation is statistically significant (*p* < 0.05).

**Table 3:** Executive function tests.

Neuropsychological Tests			Group A (*n* = 9)		Group B (*n* = 10)	
			Baseline	After 5 weeks	Baseline	After 5 weeks
Executive function tests	Intra-Extra Dimensional Shift (IED)	IED total trials (adjusted)	102.56 (46.41)	83.44 (53.74)	83.9 (29.43)	86.4 (30.77)
		IED total errors (adjusted)	26.67 (24.01)	34 (40.27)	18.3 (15.33)	18.3 (16.69)
		IED stages completed	8.56 (0.89)	10.67 (6.96)	8.8 (0.63)	8.8 (0.63)
	One Touch Stockings of Cambridge (OTS)	OTS problems solved on first choice	16.44 (2.74)	17.67 (1.94)	18 (1.41)	17.1 (3.67)
		OTS mean choices to correct (5 moves)	1.56 (0.66)	1.33 (0.25)	1.3 (0.3)	1.55 (0.98)
		OTS mean latency to first choice	9411.36 (2732.18)	10288 (5455.87)	9126.665 (3188.54)	8361.15 (4089.61)
		OTS mean latency to first choice (5 moves)	16032 (4007.63)	19483 (11173.4)	19847 (9504.19)	18191 (12756)
		OTS mean latency to correct (5 moves)	23456 (12300.8)	25025 (30267.8)	21598 (9305.57)	19581 (12484.8)
	Spatial Working Memory (SWM)	SWM between errors	31.44 (16.55)	30.22 (24.42)	38.9 (24.17)	33.2 (24.26)
		SWM strategy	27.11 (4.86)	25.56 (6.88)	31.7 (8.33)	29.5 (6.55)

The values are expressed as “mean (SD)” and none of the parameters were statistically significant in the executive function tests.
