# Supplementary material for: Allium sativum L. Improves Visual Memory and Attention in Healthy Human Volunteers
Source: Evid Based Complement Alternat Med. 2015 Aug 13;2015:103416. doi: 10.1155/2015/103416 (PMC4550798; doi:10.1155/2015/103416)
Supplement: Supplementary file 1 — Allium sativum L. (Garlic) was sliced into pieces and then crushed with a mortar and pestle to form a paste. The paste was dried under the sun and then reduced to a fine powder using a dry blender. Dried garlic powder (400mg) was then filled into empty capsules with the help of a small-scale capsule filling machine. The prepared capsules were then taken by healthy human volunteers for 5 weeks. The volunteers participated in 6 computerized neuropsychological tests of the Cambridge Neuropsychological Test Automated Battery (CANTAB) at the beginning and end of the 5 weeks study period. The results showed statistically significant variation in visual memory and attention while statistically non-significant variation was found in executive function. [file 103416.f1.docx]

**Supplementary documents**

***Allium sativum* L. Improves Visual Memory and Attention in Healthy Human Volunteers**

Sara Tasnim^1†^, Parsa Sanjana Haque^1†^, Md. Sazzadul Bari^1^**^†^**, Md. Monir Hossain^2^, Sardar Mohd.Ashraful Islam^3^, Mohammad Shahriar^3^, Mohiuddin Ahmed Bhuiyan^3^, **Muhammad Shahdaat Bin Sayeed***^3,4^

^1^*Department of Pharmacy, University of Dhaka, Dhaka-1000, Bangladesh*

*2Department of Pharmacy, Noakhali Science and Technology University, Noakhali-3814, Bangladesh*

*3Department of Pharmacy, University of Asia Pacific, Dhaka-1209, Bangladesh*

*4Department of Clinical Pharmacy and Pharmacology, University of Dhaka, Dhaka-1000, Bangladesh*

*Corresponding Author

**Brief study outcome and Graphical Abstract**

*Allium sativum* L. (AS) was sliced into pieces and then crushed with a mortar and pestle to form a paste. The paste was dried under the sun and then reduced to a fine powder using a dry blender. Dried garlic powder (400mg) was then filled into empty capsules with the help of a small-scale capsule filling machine. The prepared capsules were then taken by healthy human volunteers for 5 weeks. The volunteers participated in 6 computerized neuropsychological tests of the Cambridge Neuropsychological Test Automated Battery (CANTAB) at the beginning and end of the 5 weeks study period. The results showed statistically significant improvement in visual memory and attention. The graphical abstract is provided in the following page

**Graphical Abstract**


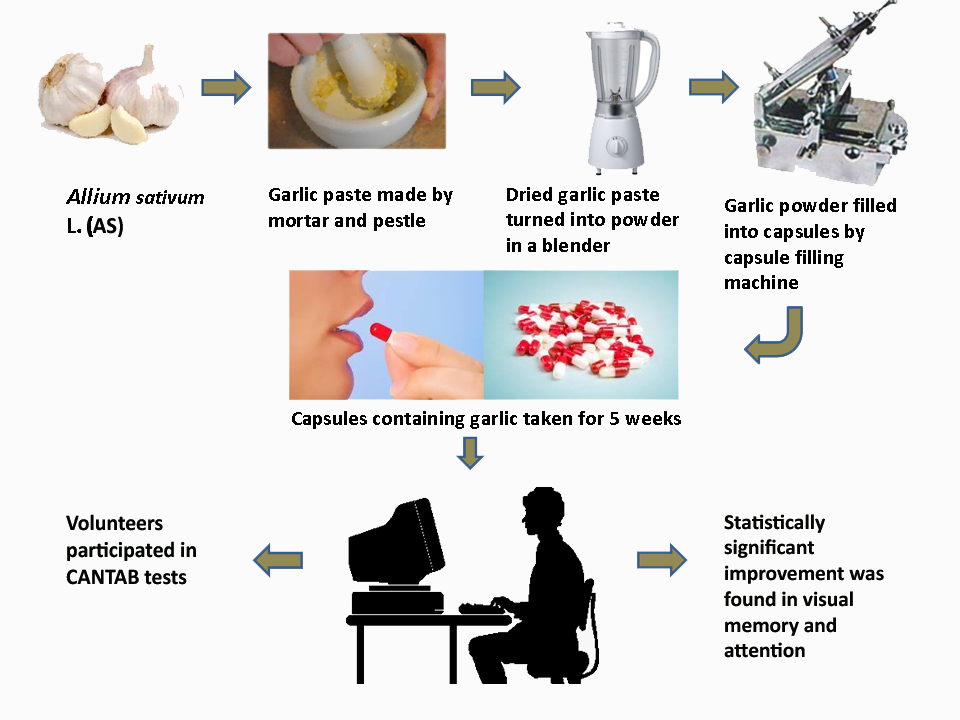


*Manuscript no.: [Evidence-Based Complementary and Alternative Medicine (eCAM):103416*
